# Effects of nitrogen deposition and phosphorus addition on arbuscular mycorrhizal fungi of Chinese fir (*Cunninghamia lanceolata*)

**DOI:** 10.1038/s41598-020-69213-6

**Published:** 2020-07-23

**Authors:** Chuyu Lin, Yaoxiong Wang, Meihua Liu, Quan Li, Wenfa Xiao, Xinzhang Song

**Affiliations:** 1 State Key Laboratory of Subtropical Silviculture, Zhejiang A&F University, Hangzhou, 311300 China; 2 Research Institute of Forest Ecology, Environment and Protection, Chinese Academy of Forestry, Beijing, 100091 China

**Keywords:** Climate-change ecology, Forest ecology, Forestry, Plant ecology

## Abstract

Nitrogen (N) deposition is a key factor that affects terrestrial biogeochemical cycles with a growing trend, especially in the southeast region of China, where shortage of available phosphorus (P) is particularly acute and P has become a major factor limiting plant growth and productivity. Arbuscular mycorrhizal fungi (AMF) establish a mutualistic symbiosis with plants, and play an important role in enhancing plant stress resistance. However, the response of AMF to the combined effects of N deposition and P additions is poorly understood. Thus, in this study, a field experiment was conducted in 10-year Chinese fir forests to estimate the effects of simulated nitrogen (N) deposition (low-N, 30 kg ha^−1^ year^−1^ and high-N, 60 kg ha^−1^ year^−1^) and phosphorus (P) addition treatments (low-P, 20 mg kg^−1^ and high-P, 40 mg kg^−1^) on AMF since April 2017, which was reflected in AMF root colonization rates and spore density of rhizosphere soil. Our results showed that N deposition significantly decreased AMF root colonization rates and spore density. In N-free plots, P addition significantly decreased AMF root colonization rates, but did not significantly alter spore density. In low-N plots, colonization rates significantly decreased under low P addition, but significantly increased under high P addition, and spore density exhibited a significant decline under high P additions. In high-N plots, colonization rates and spore density significantly increased under P additions. Interactive effects of simulated N deposition and P addition on both colonization rates and spore density were significant. Moderate N deposition or P addition can weaken the symbiotic relationship between plants and AMF, significantly reducing AMF colonization rates and inhibiting spore production. However, a moderate addition of P greatly enhances spore yield. In the case of interactive effects, the AMF colonization rates and spore density are affected by the relative content of N and P in the soil.

## Introduction

Arbuscular mycorrhizal fungi (AMF), a heterogeneous group of diverse fungal taxa and the most widespread fungal symbionts of plants, can establish mutualistic associations with the roots of over 80% of all terrestrial plant families, and plays a critical role in plant nutrient acquisition, growth, and ecosystem sustainability^1–3^. The network of AMF extraradical mycelium in the soil supports water and mineral nutrition of the host plant, especially for enhancing the supply of phosphates, by effectively absorbing and translocating mineral nutrients (e.g., N and P) beyond the depletion zones of the plant rhizosphere^4,5^. In return, plants provide carbon to AMF (as a carbon (C) source) by transferring hexose produced by photosynthesis via roots^6,7^, or in form of lipids. Typically, 5–10% of photosynthetically fixed C is allocated to the fungal partner^8^. Owing to their filamentous organization, fungi exploit diverse substrates on the basis of their nutritional strategy^9^ and significantly contribute to the uptake of soil nutrients, increase plant biomass, and improve the plant resistance to nutrient stress. Therefore, AMF have received a great deal of attention from researchers^10^. The mycorrhizal symbioses play an essential role in the N cycle by affecting processes such as organic N mineralization, biological N fixation, and N leaching, thereby increasing the N transport pathway of AMF to different forms^11^. Moreover, AMF colonization can alter the pH of the rhizosphere soil, which can activate insoluble phosphate to improve its availability, as well as promote the growth and development of the host plants by inducing the expression of the P transporter gene in plants, altering the kinetic parameters of plant nutrient absorption, and promoting photosynthetic phosphorylation.

Nitrogen deposition is a crucial issue with regard to global climate change. The increasing consumption of fossil fuels and application of agricultural fertilizers have largely enhanced the input of anthropogenic available N into ecosystems worldwide in the last few decades^12,13^. It is predicted that by the middle of the twenty-first century, global N deposition levels will appear to be twice what they were at the end of the twentieth century^14^. China is currently one of the three areas worldwide with the greatest concentration of N deposition. Particularly in the subtropical region, the most severe N deposition has reached 63.53 kg·ha^−1^·year^−1^^15,16^. Nitrogen deposition has resulted in increases in soil available N and changes in soil pH and N:P ratio. The activities of N metabolism enzymes and the accumulation of N assimilates in plants may also be altered, thereby affecting plant growth^17^. In addition, the change in soil properties and physiological function of plants will directly affect colonization, growth, N metabolism, and P uptake of AMF^18^. Studies have shown that high N levels (10 mmol·L^−1^ NH_4_C1–NH_4_NO_3_ 3:1) contributed to a significant decline in colonization rates, because excessive N inputs into the soil weaken the symbiotic synergy between AMF and host plants^19^. Furthermore, N deposition also affects plant P uptake. He et al*.*^20^ reported that the P content in the aboveground part of the plant inoculated with AMF increased with increasing N content, while the P content in the root decreased continuously.

Phosphorus is one of the most vital nutrients affecting plant growth and metabolism. However, in south China, the shortage of available P in subtropical acidic soil is particularly severe because large quantities of P remain insoluble and easily fixed. Therefore, low P stress has become one of the key factors restricting plant growth and productivity. Arbuscular mycorrhizal (AM) symbiosis plays an essential role in the adaptation and tolerance of plants to low P stress, and is the most important mechanism for improving the efficient utilization of plant P^21^. After forming the AM symbiosis, the absorption range of the symbiont expands through the symbiont mycelium, thereby greatly improving the symbionts absorption capacity for P^22,23^. Mycorrhiza can also activate insoluble P to improve the absorption efficiency of P in plants by secreting organic acid and acid phosphatase^24^. However, the availability of P in soil affects the colonization of AMF on plants, and is not conducive to the formation of mycorrhiza if out of range^25^. It has been reported by Zhang et al*.*^26^ that AMF mycorrhizal colonization rates, mycelium density, and spore numbers of corn (*Zea mays*) were significantly higher at the P50 level (50 mg·kg^−1^) than those without P addition. As the P treatment increased to 200 mg·kg^−1^ and 500 mg·kg^−1^, mycorrhizal colonization rates declined.

Chinese fir (*Cunninghamia lanceolata* (Lamb.) Hook.) is a unique tree species for afforestation and timber use in China, with a widespread natural distribution and artificial cultivation. It plays an important role in China’s artificial forests and has become a common tree species in Chinese subtropical forest ecosystems^27^. With regard to global climate change, N deposition and low P stress pose a threat to the yield of Chinese fir forests, while the requirements of a well-established AM symbiosis for induced resistance to stress is generally accepted^28^. However, the effects of simulated N deposition and P addition on AM symbiosis remains unclear, and using commercial timber species as host plants under natural field conditions remains poorly investigated. Therefore, the aim of this study was to examine the influence of N deposition and P addition on AMF by imposing simulated N deposition and P addition to the soil in a Chinese fir forest. The objectives of the study were to identify the main factors affecting the AMF mycorrhizal effect of Chinese fir trees and to demonstrate their importance for regulating and improving the utilization efficiency of soil N and P nutrients of Chinese fir trees. This will provide a theoretical basis and reference for the application of AMF for improving the development and management of the timber forest industry under the background of global environmental change.

## Results

### Root colonization rates

**Figure 1:**
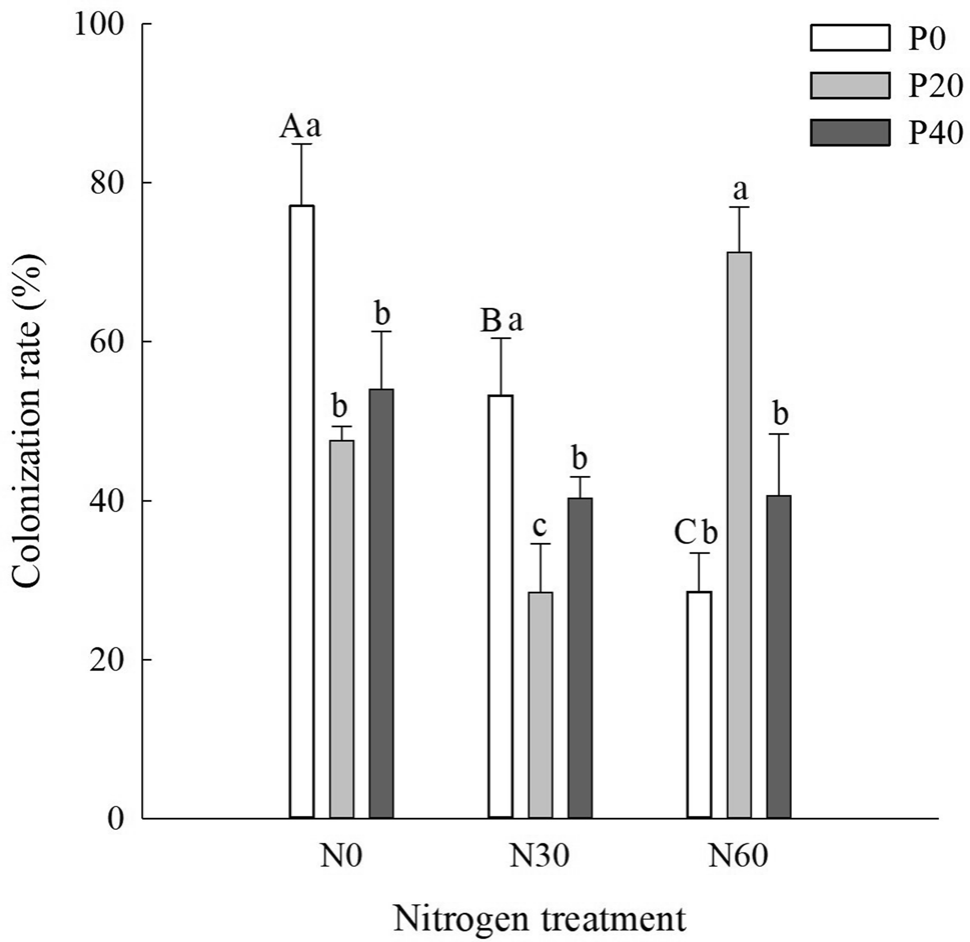
Arbuscular mycorrhizal fungi (AMF) root colonization rates of *Cunninghamia lanceolata* under nitrogen and phosphorus treatments. N0: 0 kg N∙ha^−1^∙year^−1^; N30: 30 kg N∙ha^−1^∙year^−1^; N60: 60 kg N∙ha^−1^∙year^−1^; P0: 0 mg·kg^−1^; P20: 20 mg·kg^−1^; P40: 40 mg·kg^−1^. Different lowercase letters indicate significant differences under the same simulated N deposition gradient, while different uppercase letters indicate significant differences under the same P addition gradient, *P* < 0.05. Bars denote the standard error (n = 3).

In the P-free plots, N addition reduced colonization rates, and maximum mycorrhizal colonization rates (77.1%) were evident in the N0 + P0 treatment (*P* < 0.05) (Fig. 1). Moreover, mycorrhizal colonization rates under the N60 + P0 treatment were significantly lower than that under the N0 + P0 and N30 + P0 treatment (*P* < 0.05). With the addition of P alone, colonization rates decreased with increasing additions of P. In the N30 plots, mycorrhizal colonization initially decreased and then increased with increasing additions of P, with a significant decline in colonization rates under the N30 + P20 treatment (*P* < 0.05) (Fig. 1). However, in the N60 plots, colonization rates increased under N60 + P20 treatment and decreased under N60 + P40 treatment. Compared with the N60 + P0 and N60 + P40 treatments, colonization rates of the N60 + P20 treatment increased significantly (*P* < 0.05) (Fig. 1).

### Spore density

**Figure 2:**
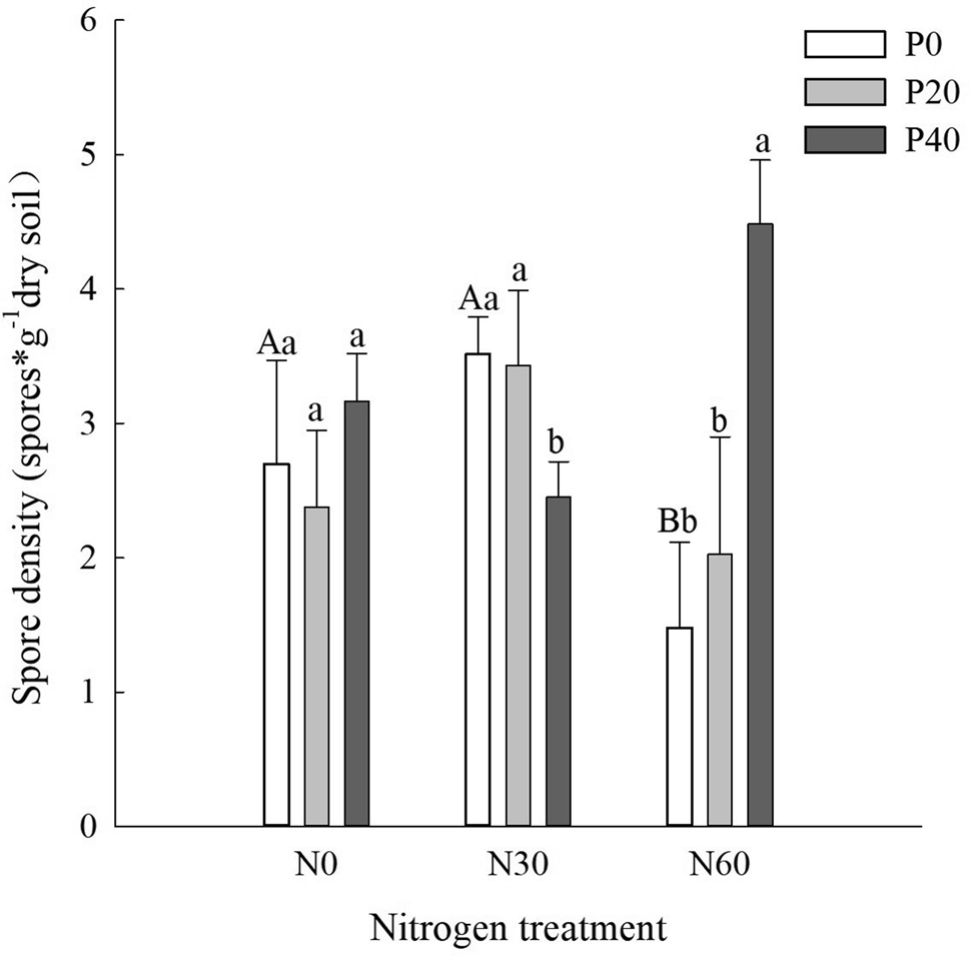
Arbuscular mycorrhizal fungi (AMF) spore density of *Cunninghamia lanceolata* under nitrogen and phosphorus treatments. N0: 0 kg N∙ha^−1^∙year^−1^; N30: 30 kg N∙ha^−1^∙year^−1^; N60: 60 kg N∙ha^−1^∙year^−1^; P0: 0 mg·kg^−1^; P20: 20 mg·kg^−1^; P40: 40 mg·kg^−1^. Different lowercase letters indicate significant differences under the same simulated N deposition gradient, while different uppercase letters indicate significant differences under the same P addition gradient, *P* < 0.05. Bars denote the standard error (n = 3).

In P-free plots, AMF spore density decreased with increases in the N level. Compared with N0 + P0 treatment, spore density under the N60 + P0 treatment decreased by 45.2% (*P* < 0.05). In the N0 plots, there was no significant variation in the spore density of AMF with increases in P addition (Fig. 2). In the N30 plots, P addition significantly reduced spore density (*P* < 0.05). Compared with the N30 + P0 treatment, spore density decreased by 27.3% under the N30 + P40 treatment (*P* < 0.05). In the N60 plots, AMF spore density significantly increased with P additions (*P* < 0.05), and AMF spore density in the rhizosphere soil under the N60 + P40 treatment was the highest (4.8/g dry soil), which was 2.26 times higher than that of N60 + P0 (Fig. 2).

### Relationship between AMF and soil properties

We determined soil physicochemical characteristics in the Chinese fir forest and made correlation analysis. AMF colonization rates were significantly negatively correlated with soil moisture (*P* < 0.05), but there was no significant correlation with the other soil physicochemical factors (*P* > 0.05) (Table 1). There was also no significant correlation between AMF spore density and soil moisture content, soil pH, available N, available P, total N, and total P (*P* > 0.05) (Table 1).

**Table 1:** Correlations between root colonization rates and spore density of arbuscular mycorrhizal fungi (AMF) and soil physicochemical characteristics.

	Soil moisture	pH	AN	TN	AP	TP
Root colonization rates	− 0.402*	− 0.118	− 0.140	− 0.185	− 0.076	− 0.154
Spore density	0.000	0.217	− 0.061	0.042	0.164	0.024

*AN* available nitrogen, *TN* total nitrogen, *AP* available phosphorus, *TP* total phosphorus.

*P < 0.05.

### Combined influence of N deposition and P addition on AMF

The two-way ANOVA analysis revealed that N deposition and P addition significantly affected root colonization rates of AMF (*P* < 0.05). In addition, the contribution of N deposition was greater than the independent effects of P additions and their interaction (Table 2). Except for P additions, both N deposition and interaction significantly affected AMF spore density in rhizosphere soil. Moreover, the contribution of the interaction was greater than that of N deposition.

**Table 2:** Two-way ANOVA indicating the effects of nitrogen and phosphorus treatments on arbuscular mycorrhizal fungi (AMF).

Factors	Source of variation	SS	*df*	MS	*F*
Root colonization rates	N deposition	19,287.87	2	9,643.94	712.25**
	P addition	451.99	2	226.00	16.69**
	N × P interaction	1,015.09	4	253.77	18.74**
Spore density	N deposition	6.82	2	3.41	4.64*
	P addition	1.34	2	0.67	0.91
	N × P interaction	17.92	4	4.48	6.11**

*SS* sum-of-squares, *df* degrees of freedom, *MS* mean square.

* *P* < 0.05 and ** *P* < 0.01, respectively.

## Discussion

### Effects of N deposition on AMF

The present study showed that the effect of simulated N deposition on AMF colonization and spore growth was dependent on the different additions of P to the soil. In the P-free plots, atmospheric N deposition increased soil N availability whereas ectomycorrhizal colonization rates decreased, which is consistent with the findings in previous studies^29^. With excessive N input into the soil, the increasing NH_4_^+^ concentration will cause mitochondrial swelling and cytoplasmic cracking in the plant’s root system. Besides, the growth and development of the host plant will be influenced since ammonium nitrogen must be assimilated in the root immediately after being absorbed by the mycorrhiza, which consumes a large amount of carbohydrates^30^. The extraradical fungal hyphae may be also poisoned, thereby the synergistic relationship of the symbionts will be affected^31^. Under the conditions of P additions, colonization rates will be reduced once soil N content has improved. According to the cost–benefit models^32,33^, the demand relationship between plants and AMF will be weakened when N and P obtained by plants is sufficient. As the mechanisms of underground nutrients change, the amount of photosynthetic products allocated to the mycorrhiza may be also reduced^34^, which makes plants become more efficient in allocating energy. In addition, the decline in colonization rates leads to a corresponding reduction in spore density. The Functional Equilibrium model^35^ indicates that plants growing in nutrient-rich soils allocate relatively more energy to stems and leaves in response to other limiting factors. In soils with sufficient N and P content, relative allocation to arbuscules, coils, and extraradical hyphae should be reduced. Colonization rates affect the capacity of AMF to confer its associated host plant with soil nutrients to obtain the C it needs for growth, which directly affects spore germination and the growth of fungal hyphae^36^. This reveals that the quantity of carbohydrates provided by the root system of the Chinese fir host was an important determinant factor in AMF spore yield^37^. The decrease of spore density can slow down the asexual reproduction of fungi, reduce the energy consumption, and help AMF survive adversity. Numerous field studies have shown that AMF colonization rates and spore density are closely related to soil factors^38^. Nonetheless, excessive soil nutrient content was generally thought to inhibit mycorrhizal symbiosis^39^. In our study, the correlation analysis showed that colonization rates and moisture content were negatively correlated at the 0.05 level. We surmise that the long precipitation period prior to sampling reduced the soil oxygen content, which is not conducive to AM symbiosis^40,41^. An alternate possibility is that AMF phylotypes can not take up soil nutrients (especially P) under the conditions with high soil moisture content, which alters the synergistic relationship between AMF and plants^42^.

### Effects of P addition on AMF

P is also an important factor affecting AMF colonization intensity and spore growth. In the N-free plots, P additions improved soil nutrients to a certain extent. As plant dependence on AMF decreased, colonization rates decreased, which is consistent with the research conducted by Zhang et al*.*^43^ It has been reported that when soil P is abundant, plants can obtain more P nutrients through their roots, which is an efficient way of energy allocating and contributes to the inhibition of the growth, extension rate, and fungal activity of AMF extraradical fungal hyphae. Thereafter, with a reduction in colonization rates, the effect of mycorrhiza on promoting P uptake and growth of plants would be reduced^44,45^. Under N30 + P0 treatment, P deficiency is relatively exacerbated by N addition to P-deficient soils, which contributes to strengthening the AM symbiosis. AMF obtain a large amount of carbohydrates from host plants, thereby enhancing the growth of symbionts and the absorption of soil P. At the same time, sporulation activity of AMF is also promoted, with the number of spores increasing to a certain extent. However, in our study, colonization rates significantly increased under the N30 + P40 treatment, which indicates that a high P gradient caused the relative deficiency in soil N. The incongruous relative content of N and P enables plants to initiate physiological mechanisms to enhance the absorption of N, thus strengthening the symbiotic relationship between AMF and plants. This provides a possible explanation for the reduction in the colonization rates under the N30 + P20 treatment, mainly owing to the relative harmonious proportion of N and P content. Under high N (N60) treatments, a relative reduction in available P caused by N deposition makes P the main limiting factor. Mycorrhizas have been considered ‘nature’s response to the law of the minimum’ because they enhance plant access to limiting belowground resources in a predictable way^46^. Arbuscular mycorrhizas have long been recognized for their importance to plant P nutrition. Thus, colonization rates greatly increased with low P additions, which promote the absorption of soil P. Nevertheless, when an excessive amount of P is added and the demand for P by host plants is adequate, the increasing P content in the plant will generate a decrease in secretion quantity or a change in secretion composition in the root system. This will have an inhibitory effect on AMF reproduction and mycorrhizal colonization^47^, thereby leading to a reduction in colonization rates under the N60 + P40 treatment. Spores are an important P pool^48^, and a moderate amount of P addition to the soil enhances spore numbers, which probably caused the significant increase in spore density under the N60 + P40 treatment. Previous studies have shown that P content in the soil is an important factor affecting the growth of AMF^49^, and the effect of AMF on improving P nutrition in plants depends strongly on the soil fertility, especially concerning the available P level in the soil^50^.

### Combined influence of N deposition and P addition on AMF

We inferred from our results that N deposition could alter the relative P content in the soil, exacerbating the effects of high P sensitivity on symbionts. It also revealed the importance of P in alleviating the N deposition stress on AMF by affecting the synergistic relationship between AMF and host. Many studies indicate that soil properties have a direct effect on the formation and development of arbuscular mycorrhiza and number of spores, and that AMF colonization rates are closely related to soil N and P^51^. In our study, two combination treatments N60 + P20 and N30 + P40 caused disequilibrium of soil nutrients. The increasing N content in the soil will stimulated more P demand for the host plants, which are relatively deficient. Hence, the root system of the plant will invest in AMF to resist adversity, which will significantly increase colonization rates. Furthermore, colonization rates of the N60 + P20 treatment were higher than that of the N30 + P40 treatment, whereas spore density was lower. This suggests that P deficiency is more likely to increase colonization rates and decrease spore-producing activity. Research has already demonstrated that decreases in the relative availability of soil P due to the large supply of N will increase the absorption and utilization of soil P through mycorrhiza, to improve plant P nutrition and balance the N:P ratio in vivo^52^. Well-structured soil nutrients can weaken the symbiotic relationship between plant and AMF, which may provide an explanation for AMF colonization rates of N60 + P20 and N30 + P40 treatments being higher than that of N30 + P20 and N60 + P40 treatments. However, AMF spore density increased significantly under the N60 + P40 treatment, indicating that under adequate soil nutrients conditions, spores are used to store nutrients and promote their continuous growth and reproduction in order to cope with an unpredictable future environment^53,54^.

## Conclusion

Under both simulated N deposition and P addition treatments, AMF colonization rates were significantly reduced, the symbiotic relationship was weakened due to a lack of C resources needed by AMF from the roots of the fir trees, and spore propagation was inhibited. N deposition and P addition improved soil nutrients, thereby weakening the demand and dependence between plants and AMF, thus threatening the symbiotic relationship. However, the relationship strengthened under low-N high-P treatments, as this caused the relative deficiency of soil N, which enhanced N absorption by Chinese firs. Under high-N treatments, a large supply of N reduced the relative availability of soil P, causing significant increases in colonization rates, while high P treatments increased spore yield. A moderate N deposition or P addition can weaken the symbiotic relationship. However, in the case of interactive N and P effects, AMF colonization rates and spore density were affected by the relative content of N and P in the soil. There are other soil physicochemical characteristics affect AMF. Soil moisture content was the main environmental factor affecting AMF colonization rates. It must be pointed out that the present conclusion was drawn from one-point sampling, and more samplings should be done for the more robust conclusion in the future study. This study provides a new approach to improving the management and productivity of Chinese fir forests under the background of global environmental change.

## Materials and methods

### Study site

This study was conducted in Gaokan village (119° 67′ E, 30° 21′ N), Lin'an District, Zhejiang Province, China. It is located on the northern margin of the subtropical monsoon climatic zone, and characterized by four distinct seasons, mild climate, and abundant precipitation. The average annual temperature of the site was 15.6 °C with an average annual precipitation of 1,420 mm, and an average of 230 frost-free days per year. The soil is classified as Ferrisols derived from granite^16^, and the terrain is comprised of low hills. Local N deposition rate is 30–37 kg·ha^−1^·year^−1^^16^.

### Experimental design

We selected 27 ten-year-old Chinese fir trees with a similar growth form and average height of approximately 3 m in the sample forest. A 3 m × 3 m independent plot was established at the center of each tree, with an interval distance of at least 2 m. Based on actual N deposition and the increasing trend in subtropical areas of China^55–57^, we set up two treatments: a low N (30 kg·ha^−1^·year^−1^) treatment (N30) and high N (60 kg·ha^−1^·year^−1^) treatment (N60). Two P addition treatments were also established after referring to relevant foreign and domestic research^58,59^: a low P (20 mg·kg^−1^) treatment (P20) and a high P (40 mg·kg^−1^) treatment (P40). Another four N and P compound treatments (N30 + P20, N30 + P40, N60 + P20, and N60 + P40) and a control group were established, with a total of nine treatments and three replicates in completely randomized blocks, with one replicate plot per treatment per block. Calcium magnesium phosphate fertilizer (main ingredients including Ca_3_(PO_4_)_2_, CaSiO_3_, MgSiO_3_, and 12–18% P_2_O_5_ ) was sprayed evenly and then thoroughly mixed into the soil with a deep plough to a 0.3 m depth on April 2017, so that the available P content in the upper layer of the soil reached to P20 and P40 levels (control group and N-only plots were also mechanically disturbed as those where P was added). Following April 2017, quantitative NH_4_NO_3_ was weighed, mixed with 0.225 L of water, and sprayed evenly from above the tree crown of each plot using an electric sprayer at the beginning of every month for 12 equal applications (over an entire year) throughout the experimental period. Each control treatment plot (N- and P-free) received 0.225 L of N-free water to control for the effects of the added water.

### Sampling

In July 2018, the distribution of the root systems of the selected Chinese fir trees was observed by the trench method^60^. Soil samples of 20–30 cm deep in the rhizosphere of the fir trees were dug in a circle (with a radius of 30 cm) with a spade at the base of each treatment plot (this was to remove topsoil to reduce experimental interference). Soil cores were sampled in four directions from each plant in each treatment plot, and mixed and sieved (2 mm mesh size) to remove stones, coarse roots and other plant residues. The soils loosely and tightly bound to the surface of roots were removed by clean tweezers and brush, and defined as rhizosphere soil samples^61^. Then, they were placed in sterile sealed bags and immediately transferred to the laboratory. Sifted and dried soil samples were stored at 4 °C in the refrigerator for determining physical and chemical properties and AMF spore density. Root samples were preserved with formaldehyde-acetic acid-alcohol (FAA) fixative (38% formalin, glacial acetic acid, 70% alcohol in a ratio of 1:1:9 (v:v:v)) prepared in advance in glass tubes with screw caps.

### Measurements

The samples of fine roots were stained with Acid Fuchsin according to a modified staining protocol^62^, and quantified by dividing the cross points with infected mycorrhizal by the total cross points, according the magnified gridline intersection method developed by McGonigle et al*.*^63^. The AMF spores were isolated from 20 g dried rhizosphere soil samples using wet sieving, decanting, and the sucrose gradient centrifugation method^64^. Spore density (total number of spores in 20 g of dry soil) was determined by counting the number of spores with a normal appearance (based on color, shape, surface condition and examination of spore contents) were counted under a compound microscope (40×) ^65^. Soil moisture content was determined by the drying method (105 ℃, 8 h), and soil pH was determined by a portable pH meter (FE20, Mettler Toledo, Switzerland) after the mixture (soil: water (w/v) ratio is 1:2.5) was shaken for 30 min. Soil available N was determined by the alkali-hydrolysis diffusion method^66^, and soil available P was extracted by the diacid method and determined by molybdenum-antimony colorimetry^67^. The content of soil N was determined by H_2_SO_4_–H_2_O_2_ extraction and semi-micro Kjeldahl method^68^, and the content of soil P was determined by H_2_SO_4_–H_2_O_2_ extraction and molybdenum-antimony colorimetry^69^.

### Statistical analysis

Statistical analysis was performed using SPSS 19.0 for Windows software (SPSS Inc., Chicago, IL, USA) with a one-way analysis of variance (ANOVA) followed by least significant difference (LSD) to establish quantitative differences between treatments. The relationship between soil characteristics, AMF root colonization rates, and spore density were tested using the Spearman’s correlation. Two-way ANOVA was performed to evaluate the combined influence of N deposition level and P addition on AMF root colonization rates and spore density. All data were tested for homogeneity of variance and normality of distribution prior to conducting the ANOVA. The data satisfied the assumption of homogeneity of variance. The images were all produced by SigmaPlot 12.5 (Systat Software Inc., San Jose, CA, USA).

## Data Availability

The data sets used and/or analyzed during the current study are available from the corresponding author on reasonable request.

## References

[1] Smith, S. E. & Read, D. J. *Mycorrhizal Symbiosis.* (Academic Press, New York, 2010).

[2] Campos-Soriano L, Segundo BS (2011). New insights into the signaling pathways controlling defense gene expression in rice roots during the arbuscular mycorrhizal symbiosis. Plant Signal. Behav..

[3] He XH, Duan YH, Chen YL, Xu MG (2010). A 60-year journey of mycorrhizal research in China: Past, present and future directions. Sci. Chin. Life Sci..

[4] Mao L, Liu YJ (2018). The eco-physiological functions of arbuscular mycorrhizal fungi: A review. Sciencepap. Online.

[5] Karasawa T, Hodge A, Fitter AH (2012). Growth, respiration and nutrient acquisition by the arbuscular mycorrhizal fungus *Glomus mosseae* and its host plant *Plantago lanceolata* in cooled soil. Plant Cell Environ..

[6] Pozo MJ, Azcón-Aguilar C (2007). Unraveling mycorrhiza-induced resistance. Curr. Opin. Plant Biol..

[7] Parniske M (2008). Arbuscular mycorrhiza: The mother of plant root endosymbioses. Nat. Rev. Microbiol..

[8] Bryla, D. R. & Eissenstat, D. M. *Respiratory Costs of Mycorrhizal Associations.* (eds. Lambers, H. & Ribas-Carbo, M.) 207–224 (Springer The Netherlands, 2005).

[9] Bonfante P, Genre A (2010). Mechanisms underlying beneficial plant-fungus interactions in mycorrhizal symbiosis. Nat. Commun..

[10] Rouphael Y (2015). Arbuscular mycorrhizal fungi act as biostimulants in horticultural crops. Sci. Hortic..

[11] Veresoglou SD, Chen BD, Rillig MC (2012). Arbuscular mycorrhiza and soil nitrogen cycling. Soil Biol. Biochem..

[12] Galloway JN (2008). Transformation of the nitrogen cycle: Recent trends, questions, and potential solutions. Science.

[13] Song XZ, Gu HH, Wang M, Zhou GM, Li Q (2016). Management practices regulate the response of Moso bamboo foliar stoichiometry to nitrogen deposition. Sci. Rep..

[14] Galloway JN, Cowling EB (2002). Reactive nitrogen and the world: 200 years of change. Ambio.

[15] Liu J (2013). Enhanced nitrogen deposition over China. Nature.

[16] Li Q (2019). Nitrogen depositions increase soil respiration and decrease temperature sensitivity in a Moso bamboo forest. Agric. For. Meteorol..

[17] Wang, J. F. & Liu, N. K. Research progress on mechanisms of atmospheric nitrogen deposition and its ecological impact. *Pollut. Control Technol.* **31**, 17–21+39 (2018).

[18] Lin JX (2015). Research progress on effects of nitrogen deposition on symbiont of plant-arbuscular mycorrhizal. Grassl. Turf.

[19] Wang, Y. N. *Physiological responses of Leymus chinens-arbuscular mycorrhizal symbiont to the interaction of nitrogen deposition and salt-alkali stress*. PhD Thesis, Northeast Forestry University (2016).

[20] He XL, Liu T, Zhao LL (2009). Effects of inoculating AM fungi on physiological characters and nutritional components of *Astragalus membranaceus* under different N application levels. Chin. J. Appl. Ecol..

[21] Hodge A (2004). The plastic plant, root responses to heterogeneous supplies of nutrients. New. Phytol..

[22] Lin SS (2013). Mycorrhizal studies and their application prospects in China. Acta Pratacult. Sin..

[23] Liu, R. J. & Chen, Y. L. *Mycorrhizology*. (Science Press, 2007).

[24] Su YB, Lin C, Zhang FS, Yang XL (2003). Effect of arbuscular mycorrhizal fungi (*Glomus Mosseae, Glomus Versiformea, Gigaspora Margarita* and *Gigaspora Rosea*) on phosphate activities and soil organic phosphate content in clover rhizosphere. Soils.

[25] Feng HY, Feng G, Wang JG, Li XL (2003). Regulation of P status host plant on alkaline phosphatase (ALP) activity in intraradical hyphae and development of extraradical hyphae of AM fungi. Mycosystema.

[26] Zhang SB, Wang YS, Yin XF, Liu JB, Wu FX (2017). Development of arbuscular mycorrhizal (AM) fungi and their influences on the absorption of N and P of maize at different soil phosphorus application levels. J. Plant Nutr. Fertil..

[27] Shi SZ (2017). Ecophysiological effects of simulated nitrogen deposition on fine roots of Chinese fir (*Cunninghamia lanceolata*) seedlings. Acta Ecol. Sin..

[28] Slezack S, Dumas-Gaudot E, Paynot M, Gianinazzi S (2000). Is a fully established arbuscular mycorrhizal symbiosis required for bioprotection of *Pisum sativum* roots against *Aphanomyces euteiches*?. Mol. Plant-Microbe Interact..

[29] Yang JY, Wang YH, Wen GS, Yi LT (2013). Effects of AM fungi and simulated nitrogen deposition on the growth and biomass accumulation of *Solidago canadensis* seedlings. Chin. J. Ecol..

[30] Marschner, H. *Marschner’s Mineral Nutrition of Higher Plants*. (Academic Press, New York, 2012).

[31] García IV, Mendoza RE (2008). Relationships among soil properties, plant nutrition and arbuscular mycorrhizal fungi-plant symbioses in a temperate grassland along hydrologic, saline and sodic gradients. FEMS Microbiol. Ecol..

[32] Johnson NC (2010). Resource stoichiometry elucidates the structure and function of arbuscular mycorrhizas across scales. New Phytol..

[33] Fitter AH (1991). Costs and benefifits of mycorrhizas: implications for functioning under natural conditions. Experientia.

[34] Pang L, Zhou ZC, Zhang Y, Feng ZP (2016). Effects of atmospheric N sedimentation on growth and P efficiency of *Pinus Massoniana* mycorrhizal seedlings under low P stress. J. Plant Nutr. Fertil..

[35] Johnson NC, Rowland DL, Corkidi L, Egerton-Warburton L, Allen EB (2003). Nitrogen enrichment alters mycorrhizal allocation at five mesic to semiarid grasslands. Ecology.

[36] Cai, X. Z. *Effects of nitrogen addition on arbuscular mycorrhizal fungi and nitrogen uptake of Chinese fir seedlings.* PhD Thesis, Fujian Normal University (2017).

[37] Bago B (2004). Differential morphogenesis of the extraradical mycelium of an arbuscular mycorrhizal fungus grown monoxenically on spatially heterogeneous culture media. Mycologia.

[38] Zhang X (2016). Correlation between physicochemical properties of rhizosphere soil and arbuscular mycorrhizal fungi in *Medicago sativa* grassland. Northern Hortic..

[39] Wang, J. *Effects of AM fungi on plant growth and community structure under different soil nitrogen fertility*. PhD Thesis, Lanzhou University (2018).

[40] Holguin G, Vazquez P, Bashan Y (2001). The role of sediment microorganisms in the productivity, conservation, and rehabilitation of mangrove ecosystems: An overview. Biol. Fertil. Soils..

[41] Ma LM, Wang PT, Wang SG (2014). Effect of flooding time length on mycorrhizal colonization of three AM fungi in two wetland plants. Environ. Sci..

[42] Sharma D, David K (2015). Moisture-a regulator of arbuscular mycorrhizal fungal community assembly and symbiotic phosphorus uptake. Mycorrhiza.

[43] Zhang, X. H. *The adaptability of arbuscular mycorrhizal fungi to different soil environmental factors*. PhD Thesis, Agricultural University of Hebei (2003).

[44] Li YF, Ju LH, Zhang LY, Xu GH (2013). Effects of AM fungi on plant growth and nitrogen and phosphorus utilization in rice/mungbean intercropping under different phosphorus application levels. Jiangsu Agric. Sci..

[45] Kiers ET (2011). Reciprocal rewards stabilize cooperation in the mycorrhizal symbiosis. Science.

[46] Read, D. J. *Mycorrhizas in Ecosystems-Nature’s Response to the “Law of the Minimum”*. (ed. Hawksworth, D.L.) 101–130 (CAB International, 1991).

[47] Sun XW (2011). Effects of eco-enviromental factors on the production and distribution of arbuscular mycorrhizal fungal spores. Acta Pratacult. Sin..

[48] Sun JH, Bi YL, Qiu L, Jiang B (2016). A review about the effect of AMF on uptaking phosphorus by host plants in soil. Chin. J. Soil Sci..

[49] Subhashini DV (2016). Effect of NPK fertilizers and co-inoculation with phosphate solubilising arbuscular mycorrhizal fungus and potassium mobilizing bacteria on growth, yield, nutrient acquisition and quality of tobacco (*Nicotiana tabacum*). Commun. Soil Sci. Plant Anal..

[50] Johnson NC, Wilson GWT, Wilson JA, Miller RM, Bowker MA (2015). Mycorrhizal phenotypes and the law of the minimum. New Phytol..

[51] Tessa C (2014). Nitrogen and phosphorus additions impact arbuscular mycorrhizal abundance and molecular diversity in a tropical montane forest. Glob. Change Biol..

[52] Gusewell S (2004). N: P ratios in terrestrial plants: variation and functional significance. New Phytol..

[53] Xia TT, Wang ZP, Zhang LC, Jing HR (2017). Effects of phosphorus concentration on growth of arbuscular mycorrhizal sweet corn. Ind. Microbiol..

[54] Qiu, J. J. *Molecular mechanism of phosphorus uptake by the interaction of arbuscular mycorrhizal fungi and maize*. PhD Thesis, Shandong Agricultural University (2017).

[55] Reay DS, Dentener F, Smith P, Grace J, Feely RA (2008). Global nitrogen deposition and carbon sinks. Nat. Geosci..

[56] Fang H, Mo J, Peng S, Li Z, Wang H (2007). Cumulative effects of nitrogen additions on litter decomposition in three tropical forests in southern China. Plant Soil.

[57] Mo J (2007). Response of nutrient dynamics of decomposing pine (*Pinus massoniana*) needles to simulated N deposition in a disturbed and a rehabilitated forest in tropical China. Ecol. Res..

[58] Chen ZY (2016). Relationship between growth and endogenous hormones of Chinese fir seedlings under low phosphorus stress. Sci. Silvae Sin..

[59] Leng HN (2009). Effects of phosphorous stress on the growth and nitrogen and phosphorus absorption of different formosan sweet gum provenances. Chin. J. Appl. Ecol..

[60] Wu JJ (2014). Analysis of soil respiration and components in *Castanopsis carlesii* and *Cunninghamia lanceolata* plantations. Chin. J. Plant Ecol..

[61] Yang YR (2015). Community structure of arbuscular mycorrhizal fungi associated with *Robinia pseudoacacia* in uncontaminated and heavy metal contaminated soils. Soil Biol. Biochem..

[62] Berch SM, Kendrick B (1982). Vesicular-arbuscular mycorrhizae of southern Ontario ferns and fern-allies. Mycologia.

[63] McGonigle TP, Miller MH, Evans DG, Fairchild GL, Swan JA (1990). A new method which gives an objective measure of colonization of roots by vesicular-arbuscular mycorrhizal fungi. New Phytol..

[64] Gerdemann JW, Nicolson TH (1963). Spores of mycorrhizal endogone species extracted from soil by wet sieving and decanting. Trans. Br. Mycol. Soc..

[65] Eom AH, Wilson GW, Hartnett DC (2001). Effects of ungulate grazers on arbuscular mycorrhizal symbiosis and fungal community structure in tallgrass prairie. Mycologia.

[66] Jackson ML (1973). Soil chemical analysis. Soil Sci..

[67] Olsen SR (1954). Estimation of available phosphorus in soils by extraction with sodium bicarbonate.

[68] Hess, T. M. *Tropical Soil Biology and Fertility: A Handbook of Methods.* (eds Anderson, J. M. & Ingram, J. S. I.) 245–245 (CAB International, 1990).

[69] Zhang, W. R., Yang, G. J., Tu, X. N. & Zhang, P. *Forestry Industry Standard of the People’s Republic of China: Forest Soil Analysis Method*. (China Standards Press, 1999).

